# Supplementary material for: Prediction of Prognosis in Pancreatic Cancer According to Methionyl-tRNA Synthetase 1 Expression as Determined by Immunohistochemical Staining
Source: Cancers (Basel). 2023 Nov 14;15(22):5413. doi: 10.3390/cancers15225413 (PMC10670752; doi:10.3390/cancers15225413)
Supplement: Supplementary file 1 [file cancers-15-05413-s001.zip › cancers-2678501-supplementary.pdf]

Supplementary Appendix

This appendix has been provided by the authors to give readers additional information regarding their work.

Supplement to: Prediction of prognosis in pancreatic cancer according to methionyl-tRNA synthetase 1 levels determined by immunohistochemical staining

Sung Ill Jang, Ji Hae Nahm, See Young Lee, Jae Hee Cho, Min-Young Do, Joon Seong Park,
Hye Sun Lee, Juyeon Yang, Jiwon Kong, Seunghwan Jung, Sunghoon Kim and Dong Ki Lee

Contents

1. Supplementary Results: page

Supplementary Figure S1: page 2

Supplementary Table S1: page 2

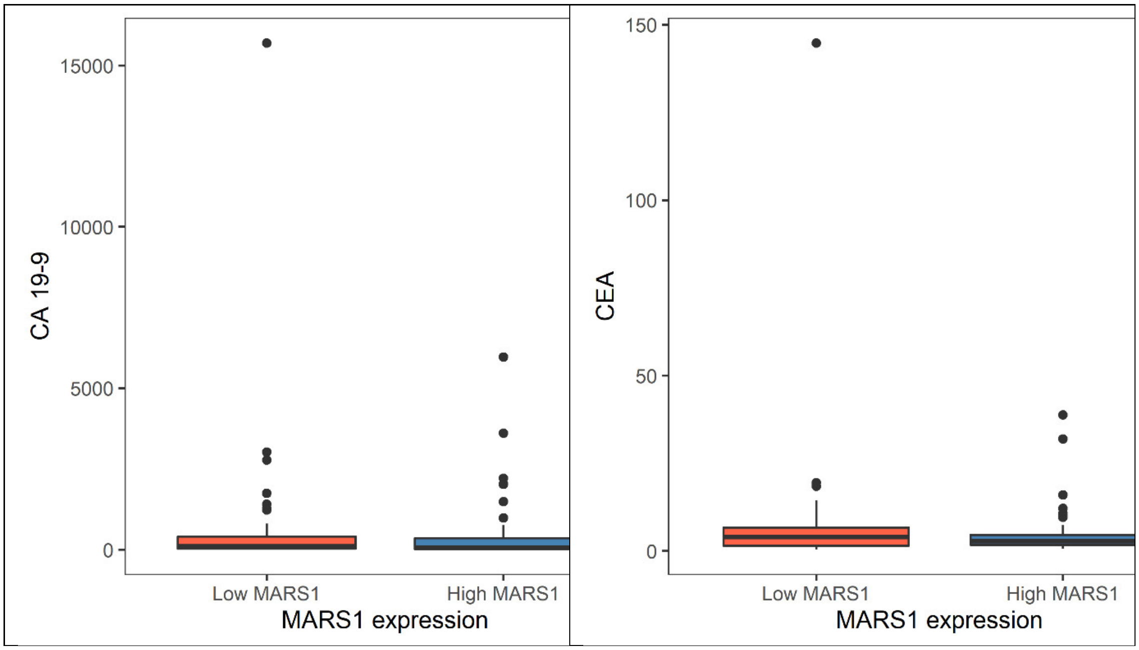

Supplementary Figure S1. CA 19-9 and CEA level analyzed using Z-score.

Supplementary Table S1 Comparison of CA 19-9 and CEA level between two group.

**Variables**	**Non-Parametric Method**		
	**Low MARS1 Expression Group (n=55)**	**High MARS1 Expression Group (n=82)**	***p*-Value**
**CA 19-9** at adm, IU/L (median(IQR))	97.80 (25.30, 408.00)	58.80 (14.50, 373.80)	0.437
**CEA** at adm, IU/L (median(IQR))	3.98 (1.40, 6.80)	2.79 (1.75, 4.60)	0.2771

MARS1, methionyl-tRNA synthetase 1; se, standard error; CI, confidence intervals.
